# Artificial Intelligence-Based Prediction of Compressive Strength in High-Performance Eco-Friendly Concrete Incorporating Recycled Waste Glass

**DOI:** 10.3390/ma19061050

**Published:** 2026-03-10

**Authors:** Ofelia Cornelia Corbu, Anca Gabriela Popa, Sepehr Ghafari

**Affiliations:** 1 Department of Structural Mechanics, Faculty of Civil Engineering, Technical University of Cluj-Napoca, 400114 Cluj-Napoca, Romania; anca.popa@mecon.utcluj.ro; 2 ERI/EUt+ Research Institute/Group, European University of Technology, European Union; 3 EUt+ Research Institute/Group, European University of Technology, European Union; 4 School of Built Environment, Engineering and Computing, Leeds Beckett University, Leeds LS2 8AG, UK

**Keywords:** machine learning, artificial neural network (ANN), compressive strength, engineered cementitious composite (ECC), high-performance concrete (HPC), waste glass powder (WGP), silica fume/microsilica (SF), waste glass aggregates (WGA), hyperparameter optimization, supplementary cementitious materials (SCMs)

## Abstract

This study investigates the application of artificial intelligence for predicting the compressive strength of a high-performance, eco-efficient engineered cementitious composite (ECC), designated mix S8-1, A. The composite incorporates supplementary cementitious materials and alternative aggregates derived from recycled glass waste. The binder system combines waste glass powder and silica fume, while the aggregate fraction includes recycled cobalt glass. An extensive experimental program involving 14 mixtures tested at 7, 28, 56, 90, and 120 days was performed to establish the reference mechanical and rheological properties. Mix S8-1, A achieved strength class C60/75 and workability corresponding to consistency class S4. To substantiate long-term performance, microstructural and chemical analyses were conducted on specimens preserved since 2011, using scanning electron microscopy (SEM) and X-ray fluorescence (XRF). The results confirmed a stable, densified microstructure, evidencing the long-term durability of the patented ECC formulation. For predictive modeling, a shallow feedforward artificial neural network with three hidden layers was developed and trained on 70 dataset entries representing mixture proportions and curing ages. Model performance was evaluated using cross-validation, achieving a coefficient of determination (R^2^) of 0.968, a mean absolute error of 1.96 MPa, and a root mean square error of 2.52 MPa. The results demonstrate that AI-based approaches can accurately predict the compressive strength of high-performance, environmentally sustainable ECCs incorporating recycled glass constituents, supporting both performance optimization and resource-efficient material design.

## 1. Introduction

Growing global concerns over climate change, carbon emissions and waste management have intensified efforts to reduce the environmental footprint of the construction sector. Cement production, a key contributor to concrete-related CO_2_ emissions, remains one of the most energy- and carbon-intensive industrial processes [1,2,3]. In response, researchers have increasingly explored circular solutions, such as the incorporation of recycled solid waste as alternative aggregate sources which can simultaneously minimize landfill disposal and preserve natural non-renewable resources [4,5,6]. Furthermore, the partial replacement of cement with supplementary cementitious materials (SCMs)—including fly ash (FA), ground granulated blast-furnace slag (GGBS), silica fume (SF), metakaolin and waste glass powder (WGP)—has proven effective in reducing the carbon intensity of concrete production while maintaining desirable mechanical and durability properties [7]. A recent study compared the embodied energy and CO_2_ emissions of with those of Ordinary Portland Cement (OPC) [8]. Embodied energy reflects the total energy consumed across a material’s life cycle—from raw material extraction to production and processing. For OPC, clinker production and subsequent grinding involve embodied energy of 4.5–5.3 MJ/kg and generate 0.73–0.91 kg CO_2_/kg [9,10]. In contrast, WGP requires only 0.06–0.14 MJ/kg and emits 0.007–0.016 kg CO_2_/kg [11]. SF, a by-product of ferrosilicon production, exhibits similarly low impacts, with embodied energy of about 0.1 MJ/kg and emissions near 0.014 kg CO_2_/kg [12,13,14,15,16]. These figures highlight that WGP and SF are energy-efficient, low-carbon SCMs that can cost-effectively reduce the environmental burden of concrete production. Environmentally friendly concretes incorporating waste glass aggregates, waste glass powder, and ultrafine silica represent innovative pathways toward sustainable construction. The use of SCMs derived from recycled glass and industrial by-products such as SF not only reduces environmental impacts but also enhances the physical and mechanical performance of engineered cementitious composites. These materials contribute to advancing sustainable construction practices by promoting resource efficiency and aligning with the waste hierarchy established in the EU Waste Framework Directive (2008/98/EC), where recycling is prioritized over disposal and incineration [17]. The development of eco-efficient construction materials in emerging economies such as Romania is driving a shift toward sustainable building practices and green design integration. Among conventional materials, concrete remains one of the most adaptable and environmentally responsive options [18], aligning with the objectives of circular economy transition policies [19,20]. The engineered cementitious composite (ECC) S8-1, A, patented in 2015 [21], was specifically developed to produce a high-performance and sustainable concrete by valorizing recycled cobalt-containing glass waste as both aggregate (WGA) and powder (WGP). To enhance mechanical behavior and matrix densification, SF was incorporated at 10% of the cement mass, supporting the composite’s advanced structural and environmental performance.

To support the present investigation on concrete incorporating waste glass aggregate, waste glass powder and silica fume, previous studies addressing the performance of glass-containing concretes were critically reviewed. A significant number of research works demonstrated that the incorporation of finely ground glass particles smaller than 0.25 mm does not induce deleterious expansion in concrete [22,23,24,25,26]. However, mixtures containing coarser glass fractions, typically within the 1.5–4.75 mm range, exhibited expansion attributed to alkali–silica reaction (ASR). Other researchers [27,28,29,30] further reported that ASR can also occur in concretes with natural aggregates due to the high silica content common to both glass and natural sand. The partial replacement of cement with SCMs such as fly ash, SF, and ground granulated blast-furnace slag has been found to effectively mitigate these reactions and reduce shrinkage [31]. Successful applications of glass-based concretes in pavements, masonry units, and precast elements were also reported [32]. Additionally, studies funded by the New York State Energy Research and Development Authority (NYSERDA) demonstrated the feasibility of using recycled glass as aggregate and additive in the production of reinforced concrete beams and precast components containing fly ash as an ASR-inhibiting agent [33,34]. A number of studies conducted worldwide [8,35,36,37,38,39,40,41,42,43,44,45,46] have emphasized the pozzolanic activity of WGP and SF, noting its significant contribution to enhancing the mechanical performance of concrete through the densification of the composite microstructure. The primary chemical mechanism involves the reaction of WGP and SF with calcium hydroxide (Ca(OH)_2_), a by-product generated during cement hydration. As hydration progresses, the released Ca(OH)_2_ reacts with the amorphous silica contained in WGP and SF to form additional calcium silicate hydrate (C–S–H) gel—the principal binding phase in concrete responsible for its compressive strength.

Other studies have focused on the sustainability of such composites [4,47]. In particular, when OPC is partially replaced by 10–15% WGP 20%, the compressive strength of engineered cementitious composites (ECC) increases by approximately 30–40% [48,49], the workability is enhanced due to the fine particle size of WGP [50] and the durability is improved, as reflected by excellent resistance to sulfate attack and alkali–silica reaction [48,49]. Similarly, replacing 10–15% of OPC with SF leads to a 50–60% increase in ECC compressive strength as a result of pore-structure refinement in the cementitious matrix, while workability remains generally high due to the pozzolanic activity of SF and durability is enhanced through significant resistance to chemical attack [51]. In the present study, the synergistic incorporation of 20% WGP and 10% SF, combined with the complete substitution (100%) of natural coarse aggregates with WGA, resulted in enhanced workability and superior mechanical strength. Furthermore, the modified concrete exhibited excellent durability performance across all evaluated parameters.

It should be emphasized that the experimental program leading to the development of ECC, S8-1, A was completed in 2011 and exhibited a high degree of innovation, combining a sustainable mixture design with superior short- and long-term mechanical and durability performance. These characteristics positioned the composite in a higher performance class at that time compared with contemporary international developments, thereby justifying the decision to file a patent application. The corresponding patent was granted in 2015 [21].

S8-1, A satisfies the consistency requirement, exhibiting an easy-to-place behavior, and meets the compressive strength criterion by attaining the strength class C 60/75, thereby qualifying as a high-performance concrete (HPC). The designation of this composite as HPC follows the definition proposed by de Larrard [52], adopted in the guidelines of the International Federation for Structural Concrete (fib), which characterizes high-performance concrete by a compressive strength greater than 60 MPa, measured on cylindrical specimens, and a water-to-binder ratio below 0.40. A complementary definition, attributed to the Prestressed Concrete Institute (PCI) and reported in earlier research works [53,54], describes HPC as concrete produced with or without silica fume, having a water-to-cement ratio lower than 0.38, a compressive strength exceeding 55 MPa and a permeability at least 50% lower than that of conventional concrete.

To substantiate the macro-scale evolution of compressive strength and other properties of the engineered cementitious composites (ECCs), particularly mixture S8-1, A, micro- and nano-scale investigations (SEM, XRF) were conducted to identify the chemical compounds associated with mineral formation and the hydration products of the ternary binder matrix. These results confirm the pronounced pozzolanic activity of SF and waste glass powder, both characterized by a high SiO_2_ content. This promotes the formation of additional calcium silicate hydrate phases in the hardened state. Owing to its very large specific surface area relative to OPC and WGP, SF provides the most significant contribution to the development of compressive strength and the enhancement of other hardened-state characteristics.

Artificial intelligence (AI) and machine learning (ML) techniques have emerged as powerful tools in materials engineering for modeling the complex relationships between mixture parameters and mechanical performance. Traditional regression-based or empirical approaches are often inadequate for capturing the nonlinear interactions among constituents in advanced concretes incorporating SCMs or recycled waste products. On the other hand, Artificial Neural Networks (ANNs) have demonstrated a strong capability to predict key mechanical properties, including compressive strength, tensile strength, and fracture energy even when available experimental datasets are relatively limited. By integrating AI-driven predictive modeling with targeted experimental programs, the development of sustainable, high-performance concrete mixtures can be substantially accelerated, thereby reducing dependence on costly and time-consuming laboratory testing [55,56,57]. Similarly, these techniques have been increasingly used to predict mechanical performance in cementitious and asphalt materials, offering improvements over conventional regression for systems with nonlinear mix–property relationships. Ensemble and kernel methods such as random forest and support vector regression provide strong baselines for compressive strength prediction under modest data regimes, while neural models can capture multivariate effects of binder composition, water ratio, and curing age with high accuracy [55,58,59,60]. Recent studies applying ML to eco-friendly mixtures highlight its suitability for recycled constituents, including waste glass powder, and demonstrate practical gains for sustainable mix optimization [55,61,62]. In parallel, AI has advanced road-engineering problems such as low-temperature fracture and mixed-mode crack growth, showing that data-driven models generalize well across specimen ages and formulations when validation is carefully controlled [59,60]. Building on this evidence, the present work couples a four-stage experimental program for an OPC + WGP + SF system with a shallow ANN and a rigorous evaluation protocol (held-out testing, grouped cross-validation by mixture, benchmarking against SVR and random forest, and noise sensitivity), thereby contributing an accurate and robust AI surrogate for eco-concrete mix design.

Therefore, in addition to the experimental investigations, this study aims to develop and evaluate optimized ANN models for predicting the compressive strength of high-performance eco-friendly concrete incorporating recycled waste glass and silica fume. The combined experimental and computational approach demonstrates how AI can be effectively applied to support mix design optimization and enhance the sustainable development of next-generation cementitious materials.

Building on the four-stage mix design and age-dependent testing, this study employs an ANN to map mixture composition and curing age to compressive strength and to provide a practical, data-driven tool that complements experimental design. The contribution lies in coupling a ternary OPC + WGP + SF high-strength ECC with a carefully validated ANN tailored to small datasets, including grouped cross-validation by mixture, a noise sensitivity analysis, and benchmarking against standard predictive models.

## 2. Materials and Methods

The mixtures designed in the practical research were made in four stages. Stage I, consisted of the development of several trial mixes, with various values of the Water/Cement (W/C) ratio and superplasticizer dosages. The result of this first step was reference mix S7, which meets strength and workability criteria proposed. Stage II consisted of developing control mixtures marked M and Mr. These were necessary to compare the results obtained on the S7 mixture and with the following mixtures that will be developed in stages III and IV. In the M mixture, crushed quarry aggregates are used, while the Mr mixture uses crushed river stone as aggregates. Stage III consisted of the development of 3 mixes, namely S8-1, S8-2 and S8-3. Stage IV consists of the development of two new mixes: S8-1, A and S8-1, B based on the results obtained for mixes S7 and S8-1. Of the two designed mixtures, S8-1, A meets the consistency criterion, being an easy-to-pour concrete, as well as the compressive strength criterion, reaching the level of strength class C 60/75, being a high-performance concrete (HPC). The next stage of this work is represented by the approach and application of artificial neural network (ANN) modeling for ECC, S8-1, A.

### 2.1. Materials

#### 2.1.1. The Ternary Binder (OPC + WGP + SF)

The main binder used in the designed mixtures was Portland cement CEM I 52.5R, in accordance with the harmonized standard SR EN 197-1 [63], with a strength class of 52.5 MPa and high early strength. In order to reach high strength classes for the composite, it is necessary to use a high-class cement. This cement was also used due to its low alkali content, reducing the possibility of alkaline-aggregate and alkaline-silica reactions [64,65]. The manufacturer reports that the grinding fineness of this OPC was 4560 ± 120 cm^2^/g, with a sulphate (SO_3_) content of 3.4 ± 0.4%. The physical and mechanical characteristics of cement are found in Table 1.

The ternary binder was made of CEM I 52.5 R cement and two SCMs, namely SF and WGP. SF and WGP are pozzolanic materials, chemically activated during cement hydration. In European standards, respectively in the harmonized Romanian ones, these SCMs are also called “Type II Additives” in accordance with SR EN 206 + A2 [69], being taken into account in the composition of the concrete designed in accordance with the concrete norm NE 012-1 [70].

**Figure 1:**
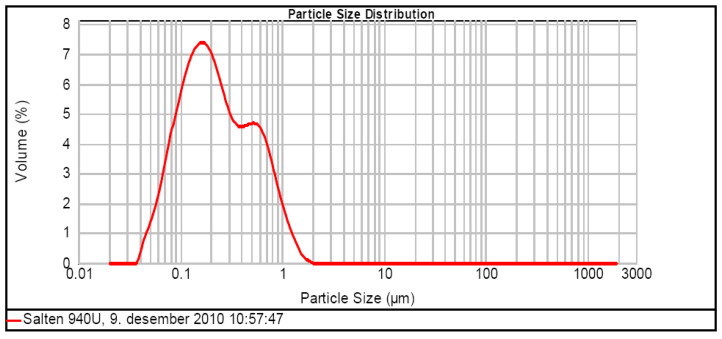
Particle size distribution of SF [71].

SF Type II Additive (pozzolanic or latent hydraulic) was an ultra-fine MICROSILICA silica supplied by BASF Construction Chemicals (Ludwigshafen, Germany). The experimental program used the Grade 940-U variant, hereinafter referred to as “silica fume”, which is an ultra-fine, non-densified silica, with an apparent weight of 250–300 kg/m^3^, with a specific surface area of 16.4 m^2^/g, a SiO_2_ content > 90% and grain sizes between 0.01 and 0.5 μm. The SF particle distribution is shown in Figure 1.

**Figure 2:**
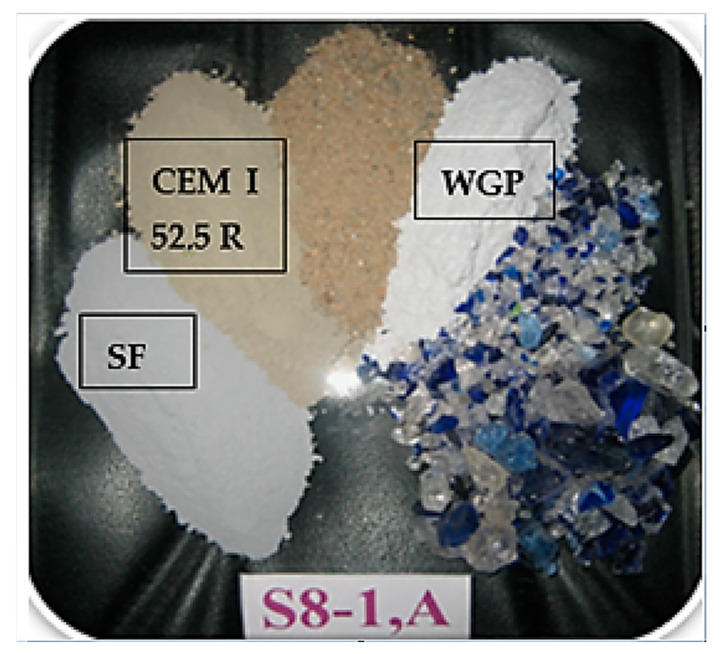
The ternary binder (OPC + WGP + SF) of the dry mixture of S8-1, A.

The WGP used to make the mixtures was of size < 0.125 mm, also used in research [65] where 100% passage through the 0.125 mm sieve can be seen. In Figure 2, the ternary group (OPC + WGP + SF) in the dry mixture of S8-1, A can be observed, which became representative at the end of the research.

**Figure 3:**
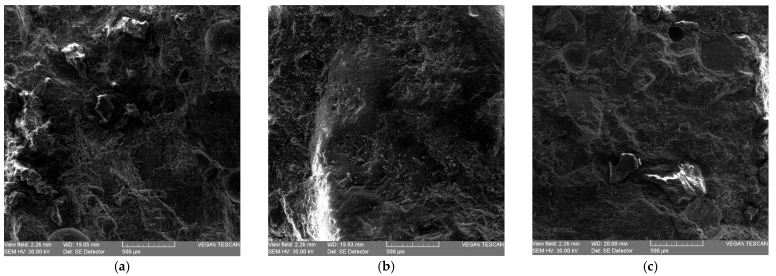
SEM Microstructures of the mixes: (**a**) OPC_M standard mixture; (**b**) OPC-10% WGP; (**c**) OPC-20% WGP [72].

In a complementary study [72], a reference cement mortar and modified mixtures incorporating SF were prepared with 10% and 20% cement replacement, respectively. Through scanning electron microscopy investigations performed on fragments of specimens previously tested in compression, the progressive consumption of fine glass particles in pozzolanic reactions initiated during cement hydration in the mixing water was observed. These phenomena are illustrated by SEM micrographs of the WGP-containing mortar composites presented in Figure 3 [72]. The mixtures with 10% replacement exhibited very good compressive strength, surpassing the reference mortar (46.7 MPa) in both the WGP-modified mixture (48.8 MPa) and the SF-modified mixture (69.2 MPa), while the mixture with 20% SF replacement also achieved a high compressive strength of 67.2 MPa. All tests were carried out on 40 mm × 40 mm × 40 mm specimens.

Through X-ray fluorescence (XRF) analysis (performed by a specialized laboratory) on cement, ultrafine silica, waste glass powder, and river sand, the chemical composition of these raw materials were obtained and are presented in Table 2.

**Figure 4:**
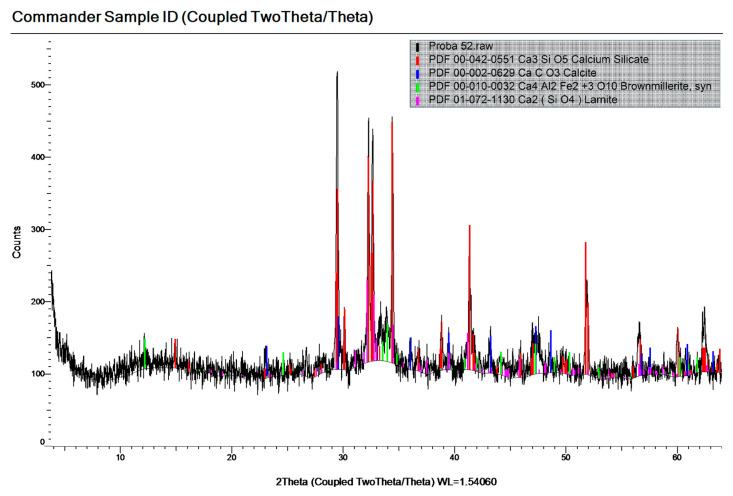
The mineralogical composition of CEM I 52.5 R.

**Figure 5:**
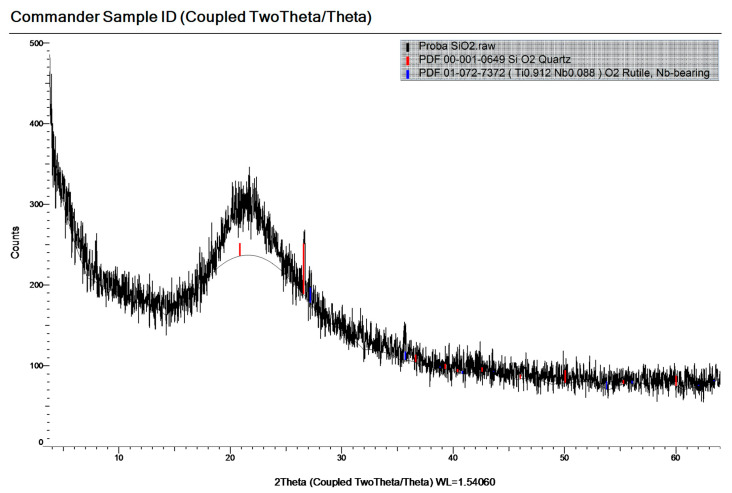
The mineralogical composition of SF.

**Figure 6:**
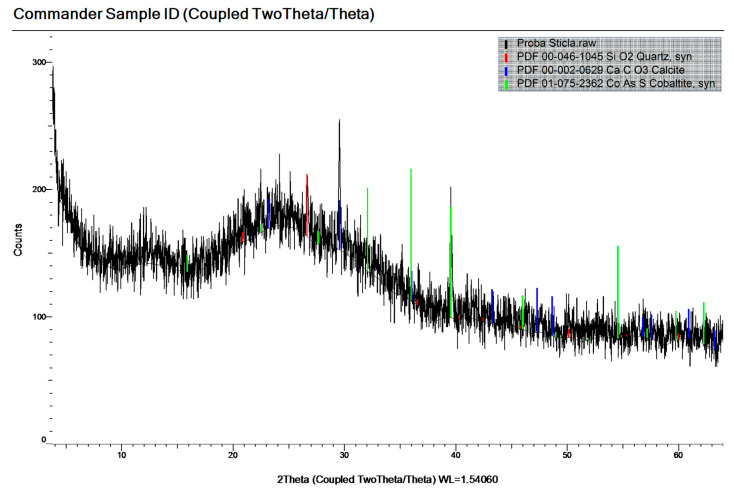
The mineralogical composition of WGP.

Figure 4, Figure 5 and Figure 6 show the mineralogical compositions of OPC, SF and WGP, respectively. The components can be found in the legend of the graph of each figure.

The main mineral phases of OPC were Calcium Silicate, Calcite, Brownmillerite, Lamite. Those of the SF were Quartz, Rutile, Nb-bearing. The corresponding mineral phases of the WGP were Quartz, Calcite, Cobaltite.

#### 2.1.2. Natural and Alternative Aggregates

**Figure 7:**
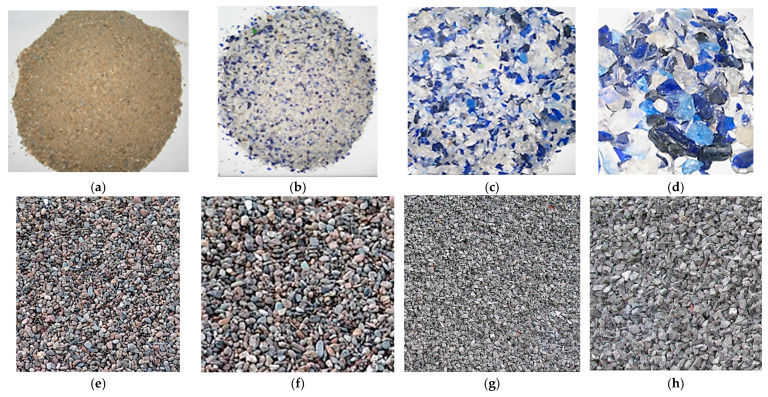
Types of aggregates used in engineered concrete mixes. (**a**) Natural river sand, Natural River Aggregate (NRA) for all main mixes 0/4 mm; (**b**) Alternative aggregate from recycled glass waste (WGA) 0/4 mm for ECCs, S8-2 and S8-3; (**c**) WGA 4/8 mm (gravel); (**d**) WGA 8/16 mm. (**e**) CRA 4/8 mm for ECC, M (gravel); (**f**) CRA 8/16 mm for ECC, M; (**g**) Crushed aggregates/Chippings (CAC) 4/8 mm for ECC, Mr; (**h**) CAC 8/16 mm for ECC, Mr.

The materials introduced into the designed mixtures are shown in Figure 7 and their physical characteristics are summarized in Table 3.

Grade 0/4 mm is considered fine sand-type aggregate and 4/8 mm and 8/16 mm are considered coarse gravel aggregate.

For the Mr control mixtures, crushed river aggregates (CRA) with the dimensions of 4/8 mm and 8/16 mm were used. For the control mixtures M, crushed quarry aggregates (CAC)/Crushed aggregates/Chippings (CAC) were used.

#### 2.1.3. Water and Superplasticizer Admixture

The water used for the concrete mixtures was from the city’s drinking water circuit. The water/cement ratio for the mixtures was 0.35 except for the final mixtures where it is slightly increased to 0.36. The high-range water-reducing superplasticizer admixture based on polycarboxylates comes from BASF Chemical Company (Ludwigshafen, Germany) and is called Glenium ACE 30. It was used at a percentage of 2% of the reference cement quantity, with exceptions made for the final mixtures: those in Stage IV, respectively, S8-1, A and S8-1, B at 2.50%. The data is summarized in Table 4.

### 2.2. Methods

#### 2.2.1. Stages in the Evolution of Design ECCs_The Developed Models Demonstrative

The mix proportions considered in this research are shown in Table 4. All SCMs calculations were related to the initial cement quantity of 465 kg/m^3^. WGP in the composition of the mixtures, substituted 20–30% of the mass of cement in the control mixture (465 kg/m^3^) and 10% SP (46.5 kg/m^3^) is an additional quantity to the amount of binder. Design parameters for concrete mixtures are in accordance with the normative [69,70].

#### 2.2.2. The Types of Determinations and Standardized Working Method for the Characteristics Reported in This Paper

Slump and compressive strength are the characteristics developed in the work.

##### Determinations of Fresh Properties of Concrete

- Consistency using the slump test (mm), in accordance with SR EN 12350-2 [73]

The slump cone has a height of 300 mm and the difference between this and the cone formed by the concrete is measured as to the slump value. Three determinations are made for each fresh mix.

##### Determinations of Hardened Properties of Concrete SR EN 12390-1 [74]

- Compressive strength (fcm) in accordance with SR EN 12390-2 [75], SR EN 12390-3 [76], SR EN 12390-4 [77].

A minimum of six cubic specimens measuring 150 mm × 150 mm × 150 mm were considered. The samples were placed in water (20 ± 2 °C) until the testing age.

- Flexural strength (fct,fl) in accordance with SR EN 12390-5 [78].

A minimum of three prismatic specimens measuring 150 mm × 150 mm × 600 mm were considered.

- Splitting tensile strength (fct,sp) in accordance with SR EN 12390-6 [79].

A minimum of three cubic specimens measuring 150 mm × 150 mm × 150 mm were considered.

- Modulus of elasticity (MOE) of concrete SR EN 13412 [80].

A minimum of three prismatic specimens measuring 100 mm × 100 mm × 300 mm were considered.

- Shrinkage measured up to 120 days of age with the Huggerberger deformometer with a measurement accuracy of 0.0001″/10″ [81].

A minimum of three prismatic specimens measuring 100 mm × 100 mm × 300 mm were considered.

##### Durability Properties of Concrete

- Loss of strength after 100 freeze–thaw cycles in accordance with SR 3518 [82].

A minimum of 12 cubic specimens measuring 150 mm × 150 mm × 150 mm were considered.

- Abrasion resistance in accordance with SR EN 1338 [83].

A minimum of six cubic specimens measuring 71 mm × 71 mm × 71 mm were considered.

- Carbonation depth determination according to SR CR 12793 [84].

A minimum of two cubic specimens or prism fragments with dimensions of 150 mm × 150 mm were taken into account.

- Permeability at 12 atm in accordance with SR EN12390-8 [85].

A minimum of three cubic specimens measuring 150 mm × 150 mm × 150 mm were considered.

#### 2.2.3. Optical Microscopy and Powder X-Ray Diffraction (PXRD) Method for Qualitative Analysis of Crystalline Constituents

##### The Mineralogical Composition of ECC, S8-1, A with Powder X-Ray Diffraction Method (PXRD)

X-ray diffraction (XRD) was performed on S8-1, A, using a Bruker D8 Advance diffractometer (Bruker, Billerica, MA, USA) with Cu Kα radiation (λ = 1.541874 Å), a 0.01 mm Fe filter, and a one-dimensional LynxEye detector at the Department of Geology, Babeș-Bolyai University (Cluj-Napoca, Romania). For this X-ray analysis, fragments from specimens S1-8, A were ground to powder sizes to obtain an overview of the mineralogical composition.

##### Optical Microscopy with Polarizing Light

For this analysis, thin sections of the investigated concrete sample S8-1, A were prepared and one-Nikon Optiphot T2—Pol (Nikon, Minato City, Japan) was used for optical studies (textural and compositional) at the crossed and parallel polars (microscope conditions), respectively, as well as for taking photographs.

#### 2.2.4. Artificial Neural Network (ANN) Modeling Approach

Artificial Neural Networks (ANNs) were used in this study as a predictive tool for estimating the compressive strength of the designed concrete mixtures. Since the number of mixture designs was limited to 14, each compressive strength value measured at different curing ages (7, 28, 56, 90 and 120 days) was considered as an individual data point. By treating age-dependent strength development separately, the total dataset expanded to 70 samples. This approach has been applied in previous studies on concrete strength prediction where curing age plays a decisive role in strength gain [86,87].

##### Input and Output Variables

The ANN models were developed using mixture composition and curing age as input features. The main mixture parameters included the cement content, SF, WGP, type of aggregates, water-to-binder (W/B) ratio, and dosage of the superplasticizer. The curing age was also included as an input variable to account for the progressive development of strength over time. The target output was the measured compressive strength (MPa) of each sample.

##### Network Architecture

**Figure 8:**
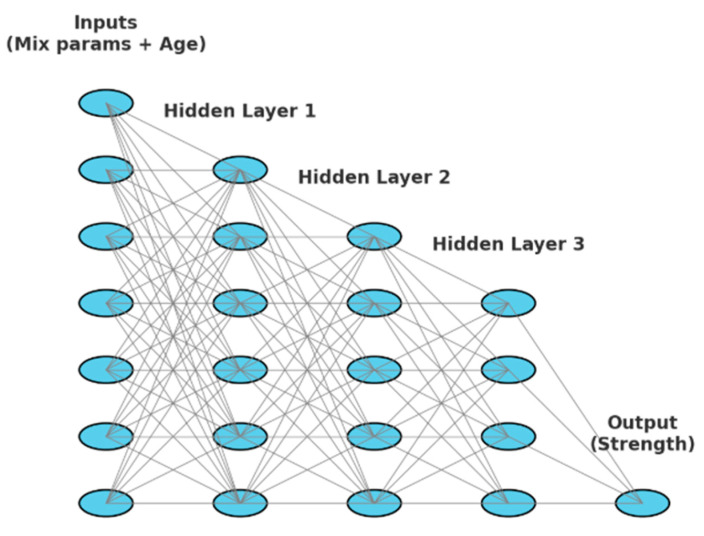
Artificial Neural Network architecture (shallow).

A shallow feedforward ANN was adopted because such networks have been demonstrated to perform effectively with small datasets, while also being less prone to overfitting compared to deeper networks [59,60,88]. The chosen ANN consisted of three hidden layers, with the number of neurons in each layer determined through trial-and-error and supported by grid search hyperparameter tuning [Table 5]. The rectified linear unit (ReLU) activation function was employed for the hidden layers, while a linear activation was used for the output layer. The architecture of the artificial neural network is shown in Figure 8.

##### Training Procedure

The models were implemented in Python 3.10.12 using the TensorFlow/Keras library. Training was performed using the Adam optimization algorithm, which combines the advantages of adaptive learning rates and momentum. The loss function was defined as mean squared error (MSE), which is a standard measure for regression-type problems such as strength prediction. The batch size and learning rate were tuned during the optimization process to improve the convergence of the network.

##### Data Preprocessing

Prior to training, all input variables were normalized to the range [0, 1]. This step ensured that variables with larger magnitudes did not dominate the learning process and that the network converged more efficiently. The expanded dataset of 70 samples was randomly split into training and testing sets, with 70 percent of the data used for training and 30 percent reserved for independent testing.

##### Validation Strategy

To further assess the robustness of the ANN, a 10-fold cross-validation strategy was applied. In this method, the dataset is divided into 10 subsets, and the network is trained on nine subsets while the remaining one is used for validation. This process is repeated 10 times until each subset has been used once for validation. Cross-validation is widely recommended for small datasets, as it provides a more reliable estimate of the model’s predictive capability than a simple train/test split [58].

##### Performance Evaluation

The predictive accuracy of the ANN was evaluated using several statistical measures: coefficient of determination (R^2^), mean absolute error (MAE), and root mean square error (RMSE). R^2^ indicates the proportion of variance in the experimental data explained by the model, while MAE and RMSE quantify the absolute and squared deviations between predicted and measured strengths. These metrics have been widely adopted in previous studies involving ANN-based prediction of concrete properties [55,87,89].

##### Benchmark Models

To contextualize ANN performance, baseline models were trained and evaluated under identical features and splits: multiple linear regression (MLR), support vector regression with an RBF kernel (SVR), and random forest regression (RF). Inputs included binder contents (OPC, SF, WGP), aggregate fractions, water and admixture dosage, density, water-to-cement ratio, water-to-binder ratio, and curing age. Features were standardized where applicable. Validation used a held-out test split and grouped cross-validation by mixture (8 folds) to prevent any ages from a held-out mixture leaking into training. Performance metrics were reported as R^2^, MAE, RMSE (as pointed out in Section Benchmark Models), and as mean ± standard deviation for cross-validation.

##### Noise Sensitivity

To assess robustness to small input perturbations, zero-mean Gaussian noise was added to the standardized input features and performance was re-evaluated using grouped cross-validation by mixture. Noise levels were σ ∈ {0.01, 0.02, 0.03} on the standardized scale. For each σ, grouped CV was repeated multiple times with fresh noise realizations and the mean ± SD of R^2^, MAE, and RMSE was reported together with changes relative to the baseline (no noise).

## 3. Results

Building on the four-stage mix design and age-dependent testing, this study em-ploys an ANN to map mixture composition and curing age to compressive strength and to provide a practical, data-driven tool that complements experimental design.

Also, to confirm the stable structure of the selected ECC, S8-1, A, microstructural investigations and compositional characteristics of it are presented, through qualitative analysis of the crystalline constituents.

### 3.1. Slump

**Figure 9:**
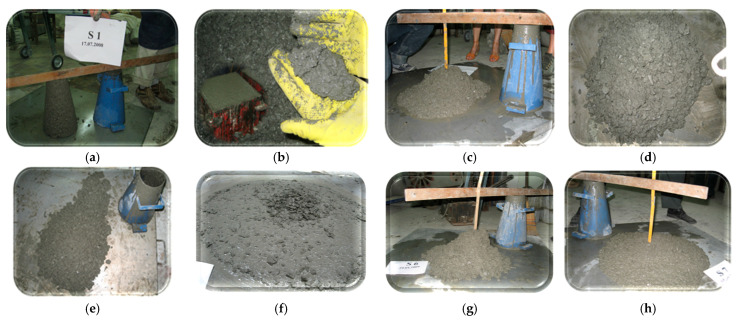
The slump for Stage I mixtures: (**a**,**b**) Mixture S1, with the slump value is 0, the mixture after vibration shows cohesion; (**c**) Mixture S2, “Shear slump”, with classification in the compaction class S5—210 mm; (**d**) Mixture S3, “Shear slump” S4—200 mm; (**e**) Mixture S4, “Shear slump” S4—200 mm; (**f**) Mixture S5, slump with central agglomeration of aggregates, S5—260 mm; (**g**) Mixture S6, “Shear slump” S4—165 mm.; (**h**) Mixture S7 with S5_—_255 mm. The slump cone has a height of 300 mm and the difference between this and the cone formed by the concrete is measured as to the slump value.

**Figure 10:**
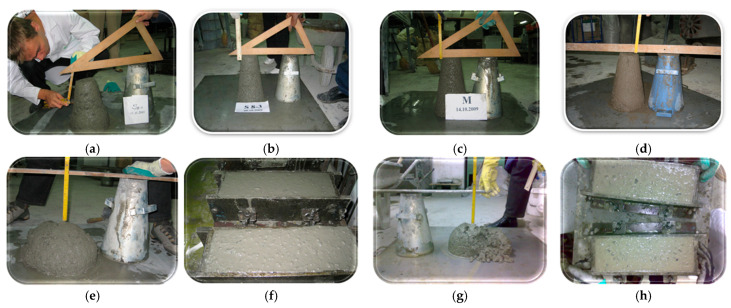
The slump for Stage II–IV mixtures: (**a**) Mixture S8-1, compaction class S2 = 53 mm; (**b**) Mixture S8-3, Cone of slump left standing, without slump; (**c**) Mixture M, Cone of slump left standing, without slump; (**d**) Mixture Mr, Cone of slump left standing, without slump; (**e**) Mixture S8-1, A, compaction class S4—188 mm; (**f**) Mixture S8-1, A, concrete appearance after vibration compaction; (**g**) Mixture S8-1, B, S4—165 mm, slump followed by collapse called “Shear slump”; (**h**) Mixture S8-1, B, concrete appearance after vibration compaction.

In Stage I, the only mix accepted as easy-to-place concrete was S7, shown in comparison with the other designed mixes, in Figure 9h. For mixes from Stages II–IV, their slump is shown in the images in Figure 10.

### 3.2. Compressive Strength of ECCs Mixtures and Other Characteristics of Durability

Table 6 shows the values of the hardened characteristics of the ECCs from S7, S8-1, S8-2, S8-3, S8-1, A, S8-1, B, M and Mr. The performance of ECC, S8-1, A is observed for all the characteristics presented in Table 6.

### 3.3. Optical Microscopy and Powder X-Ray Diffraction (PXRD) Method for Qualitative Analysis of Crystalline Constituents

#### 3.3.1. The Mineralogical Composition of ECC, S8-1, A with Powder X-Ray Diffraction Method (PXRD)

**Figure 11:**
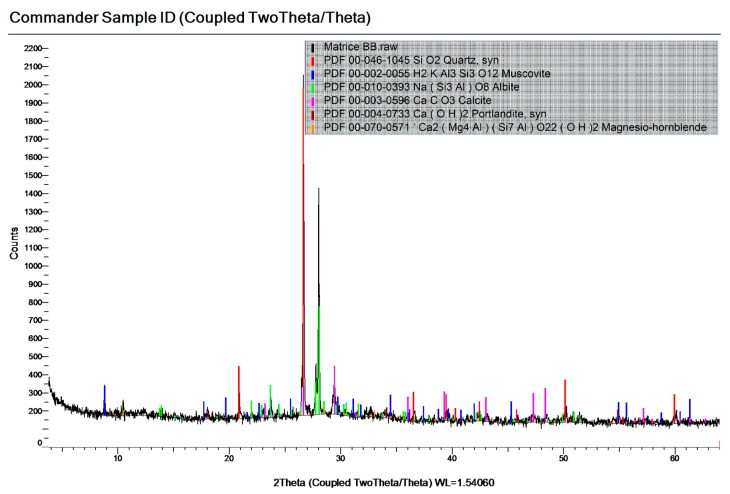
The X-ray spectra of the sample S1-8, A with the typical line for Quartz (Qz), Muscovite, Albite, Calcite, Portlandite and Magnesio-hornblende.

Minerals are formed during cement hydration and pozzolanic reaction. X-ray investigation performed on the sample from mixture S8-1, A, shown in Figure 11, indicated the presence of mineral phases originating from aggregates (Quartz, Muscovite, Albite), as well as newly formed minerals, such as Portlandite (Ca(OH)_2_) and Calcite (CaCO_3_).

#### 3.3.2. Optical Microscopy with Polarizing Light

**Figure 12:**
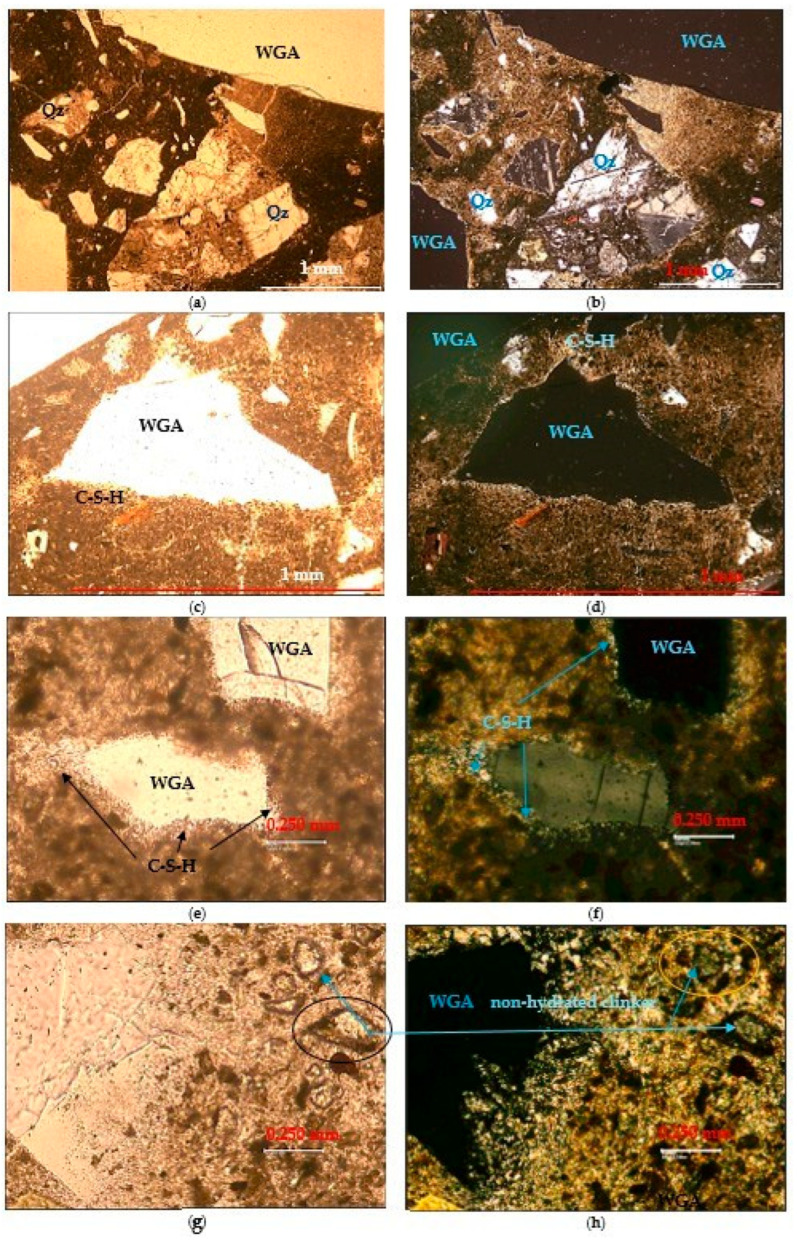
Microscopic image of the sample S8-1, A at parallel pollars (on the left side) and crossed pollars (on the right side): (**a**,**b**) WGA, CRA, CAC, Quartz (Qz), (**c**,**d**) light formations around the aggregates ((C-S-H)-hydrated calcium silicates); (**e**,**f**) light formations around the aggregates (C-S-H); WGA; (**g**,**h**) non-hydrated clinker.

In Figure 12, images obtained by exposure to polarizing light are presented to observe the anisotropic (crystallized) state of the S8-1, A engineering cementitious concrete composite. In this analysis, a microscopic study with polarizing light was performed on thin strips taken from the ECC, S1-8, A specimen, which highlights certain properties. The microscope conditions are 1 Nicol/one nicol polar (left images) and Nt/crossed polar (right images). It can be observed that all natural and glass aggregates are crystallization support for minerals in the dense matrix. The minerals form during hydration, and pozzolanic reactions, and are observed as bright formations around the aggregates, (C-S-H) as in Figure 12c–f.

In addition to portlandite, muscovite, calcium silicate hydrate (C-S-H) are visible, which are new minerals formed during hydration, and the pozzolanic reaction. The fact that there is non-hydrated clinker shows the prospect of increasing compressive strength under favorable storage conditions.

### 3.4. ANN Prediction Results and Benchmarking

The ANN provided accurate predictions of compressive strength using mixture composition and curing age. On the held-out test set, it achieved R^2^ = 0.968, MAE = 1.96 MPa, and RMSE = 2.52 MPa, as shown in Table 7 and the experimental versus predicted scatter plot. Benchmarking against MLR, SVR (RBF), and RF under identical features and splits confirmed the highest accuracy for the ANN. Under the stricter grouped cross-validation by mixture (8 folds), the ANN also retained the best mean performance with low variability (Table 8). A noise sensitivity analysis indicated only minor changes in the metrics under modest input perturbations (Table 9).

### 3.5. Noise Sensitivity

Table 9 summarizes the ANN’s performance under increasing input noise on the standardized scale. Changes relative to the baseline grouped CV (no noise) are limited and monotonic, indicating stable predictions under modest perturbations.

## 4. Discussion

Given that the main characteristics targeted in this work are workability and compressive strength for a high-performance ECCs. Having achieved a high strength for a high-density ECC, the results for durability characteristics are implicitly increased. ECC, S8-1, A enters the evaluation of predictions, having the best characteristics in the fresh and hardened state, ranking in the compressive strength class of C60/75, for a high-strength and performance concrete.

### 4.1. The Ternary Binder (OPC + WGP + SF)

SF has the role of increasing the densification of the mixture, supporting all the short and long-term properties, and WGP stands out by supporting the abrasion resistance [65] and also on the densification of the mixture the smaller the granulation. SF and WGP also act as an inhibitor of alkali-silica reaction (ASR), eliminating the possibility of cracks from contractions. Many studies by researchers have reported that WGP ground into a fine powder, as close as possible to that of cement, and due to its high SiO_2_ content, is a very effective SCM alternative, following the activation of the pozzolanic reaction [90,91,92,93,94,95,96].

### 4.2. Fresh State Characteristics of S8-1, A

The slump for the composition S8-1, A has a higher value than the composition S8-1, B due to the lower amount of WGP, which reduces the workability of the concrete by the fact that the shape of its granules is not spherical as in the case of SF. The value obtained in consistency class S4 is due to the lower water absorption, which compensates for the spherical shapes of SF but with a high-water absorption. Thus, a good packing density of the coarse WGA and good cohesion between them was achieved, without opposing the flow.

### 4.3. Compressive Strength of ECCs Mixtures and Other Characteristics of Durability

All the characteristics that were mentioned in the previous Section 3.2. confirm the influence of the use of SCMs in the composition of ECCs for the evolution of the characteristics in the hardened state in particular. The results show that the particle size and the degree of hydration of the cement together with SCMs in the matrix continued to develop up to 120 days (the last age of compressive strength testing), thus resulting in very good characteristics of the ECCs, especially of ECC, S8-1, A which is classified in strength class C60/75 as high-performance concrete, just like ECC, control M.

To establish the characteristic compressive strength fck, cub, different sources suggest the relationships shown in Table 10. The concrete class is defined based on the characteristic compressive strength fck, 28, in Table 11, which is determined on cubic specimens with a side of 150 mm or cylindrical specimens of 150/300 mm, hardened under standard conditions and tested at the age of 28 days under laboratory conditions.

Since the value Δf = 8 MPa from the relation proposed by fib (International Federation for Structural Concrete) falls within the range (6–12) MPa proposed by the concrete standard in Romania [69,70] for the production of ready-mixed concrete, this value is considered in establishing the concrete strength classes.

**Figure 13:**
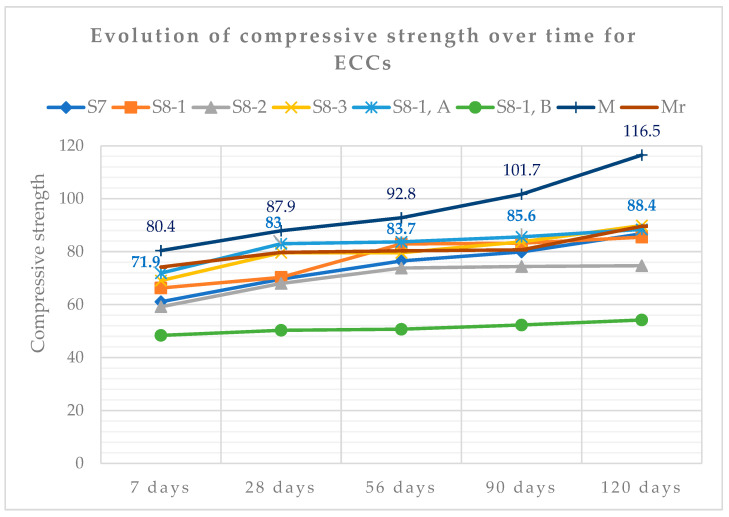
Evolution of compressive strength over time for ECCs.

Analyzing Table 6, which presents the characteristics of ECCs in the hardened state and Table 11, which presents the classification in strength classes, the analysis is reduced to ECCs, S8-1, A versus ECCs, M, classified in the same strength class and with an evolution of the characteristics, with the closest values. Due to the glass powder content of S8-1, A compared to M, which contains SF as an addition and quarry aggregates the evolution after 28 days, it does not have the same growth rate for ECC, S8-1, A, as can be seen in Figure 13. Another proof is that the nature of the aggregate skeleton and the fine powder content support the strength of the concrete.

Analyzing Table 12, it is found that there is an evolution of the compressive strengths over time of the two compositions. The evolution of the strength for ECC, M-Control, increases compared to that of composition S8-1, A between the ages of 90–120 days, with the differences in strength being 16% and 24%, respectively. For the other test ages, the difference between the strengths of ECC, M and that of ECC, S8-1, A is in the range of 6–11%. Substituting a quantity of WGP to composition S8-1, A from OPC leads to a slowdown in the evolution of the long-term compressive strength.

The E_cm_ values are those related to a higher concrete class. The class related to the M and S8-1, A mixes was C60/75 and C60, respectively, for which SR EN 1992-1-1/2004 [98] provides a value for E_cm_ of 41 GPa compared to the experimentally obtained value of 48 GPa and 54 GPa, respectively.

The ECC composite, S8-1, A, containing WGP, showed, after 120 days of monitoring, drying shrinkage values of approx. 0.30 mm/m. Their stabilization was noted after approx. 56 days from casting. Liu et al. [43] indicated that the addition of WGP inhibits the early hydration process of the mixture, delays the setting time and reduces the autogenous shrinkage (with a maximum reduction of 21.49%).

The strength loss value following the 100 repeated freeze–thaw cycles of 100 cycles, for ECC, S8-1, A was 5.54%, well below the limit value of 25%.

The permeability of ECC, S8-1, A, was at maximum 10% of the minimum allowed in the corresponding national code.

All compositions fall into the best performance class according to the performance criteria in [84], SR EN 1339:2004 [99] placing it in Class 4—mark I, which requires a volume loss < 18,000 mm^3^/5000 mm^2^.

Carbonation: 0%, it is non-existent, no ECCs have changed their basicity.

Mixture S8-1, A showed that the incorporation of the two SCMs, SF and WGP, into the mixture had beneficial influence on the durability characteristics of the concrete.

### 4.4. Optical Microscopy and Powder X-Ray Diffraction (PXRD) Method for Qualitative Analysis of Crystalline Constituents. Microstructure Characterization

The major mineral phase for SF and WGP is crystalline silica oxide (SiO_2_) while cement has calcium oxide (CaO).

Kunther, W. et al. (2017) [100] discovered in his studies that low Ca/Si ratios, in the matrix of the designed mixtures, influence the increase in compressive strengths and their evolution over time, due to the quantitative increase in C-S-H phases. In the initial stages, an additional amount of C-S-H can act as a nucleus for faster formation of the hydrated gel, leading to significant increases in compressive strength at 7 or 28 days. This effect is observed in studies using additional ultrafine SF, especially when combined with SCMs as an addition or as cement substitutes [101]. Since additional C-S-H continues to form and densify the internal network, improving the microstructure in proportion to the curing time of the ECC, it leads to a continuous development of strength [102,103].

The hydrated calcium silicate gel (C-S-H) is the fundamental phase that influences most of the mechanical properties of hardened concrete. Microscopic characterization further reveals that the complementarity and especially their interaction between WGP and SF through chemical reaction enhances the pore structure and compactness of the matrix, which is the key mechanism for enhancing the performance of WGP-UHPC [43,45].

### 4.5. Artificial Neural Network Modeling

The ANN model developed in this study provided reliable predictions of compressive strength based on mixture composition and curing age. Despite the limited number of mixture designs, the use of multiple curing ages expanded the dataset to 70 data points, which proved sufficient for training a shallow network. The predictive accuracy, expressed in terms of R^2^, MAE, and RMSE, demonstrated that the model was able to capture the nonlinear relationships between input variables and strength development.

The good performance of the ANN confirms earlier findings that shallow networks are effective when working with relatively small experimental datasets. Yeh [86] first demonstrated that ANN models could successfully predict the strength of high-performance concrete (HPC) with limited data. Topçu and Sarıdemir [87] also reported accurate predictions of fly ash concretes using ANN and fuzzy logic. More recently, Behnood and Golafshani [55] employed ANN models for concretes containing waste foundry sand and achieved similar levels of predictive capability. In line with these works, the present study shows that ANN can also be applied to eco-friendly concrete mixes incorporating WGP and SF.

An additional observation is that the incorporation of curing age as an input significantly improved model performance. This agrees with the work of Chou and Pham [58], who emphasized that time-dependent strength development must be explicitly included for accurate prediction. In the current study, the ANN model successfully differentiated strength evolution across 7 to 120 days, capturing both the early-age and long-term strength gain of the mixtures.

The results are also consistent with our previous research on asphalt concrete mixtures, where a shallow three-layer ANN achieved a higher coefficient of determination than a deeper architecture [104]. Together, these findings support the conclusion that relatively simple ANN models are well-suited for experimental datasets in civil engineering, where the cost of material testing often limits the number of samples.

The benchmarking confirms that the proposed ANN provides the most accurate predictions among the tested techniques. On the held-out test set, the ANN achieved R^2^ = 0.968, MAE = 1.96 MPa, and RMSE = 2.52 MPa, outperforming random forest, SVR, and multiple linear regression under identical conditions (Table 7). Under the stricter grouped cross-validation by mixture, the ANN maintained the highest mean accuracy (R^2^ = 0.920 ± 0.030, MAE = 2.40 ± 0.40 MPa, RMSE = 3.10 ± 0.50 MPa) with limited variability (Table 8). The noise sensitivity analysis showed only small, monotonic changes in metrics for σ ≤ 0.03 (Table 9), indicating robustness to modest input perturbations.

**Figure 14:**
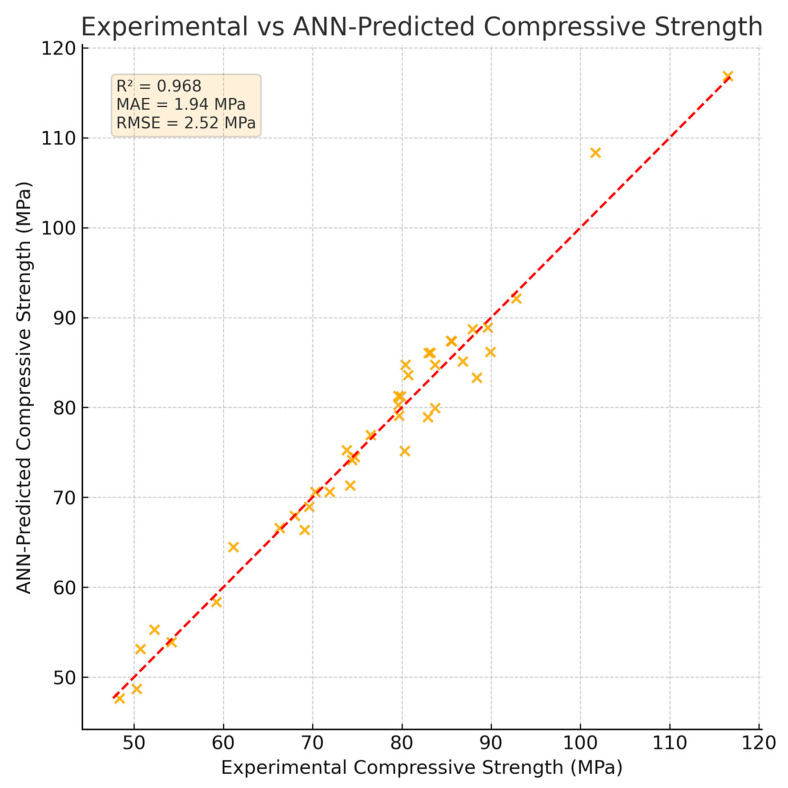
Experimental versus ANN-predicted compressive strengths for all mixtures and ages (*n* = 40). The dashed line indicates perfect agreement. The shallow feedforward ANN (three hidden layers) achieved R^2^, MAE, and RMSE as reported on the plot. Experimental values are from the present study.

Although the dataset size was constrained, the application of cross-validation ensured robust model evaluation. This approach reduced overfitting and provided more generalizable results, confirming that ANN can serve as an efficient predictive tool even under data limitations. Importantly, the ANN predictions aligned well with the experimental compressive strength values (Figure 14), highlighting the model’s potential as a supplementary design tool for developing sustainable concrete mixtures.

Artificial intelligence surrogates such as the ANN used here provide accurate macroscopic strength predictions, but a complementary mesoscale viewpoint can clarify mechanisms in heterogeneous ECCs with recycled constituents. Recent mesoscale phase field and finite element studies explicitly represent aggregates, inclusions, and interfacial transition zone to capture localized stress fields, damage initiation and propagation, and ITZ degradation, thereby linking constituent morphology and stiffness to observed macroscopic response. Representative works include phase field modeling of rubber reinforced concrete that reproduces fracture evolution across rubber contents, mesoscale analyses of metaconcretes that resolve core-coating-ITZ interactions under compression, and phase field frameworks for recycled aggregate concrete that track crack paths and damage in mixed old–new mortar systems. In this context, integrating mesoscale simulations with our ANN would enable a mechanism aware workflow in which WGP and SF effects on paste densification and ITZ stiffening are quantified through mesoscale indicators, while the ANN provides fast, data driven prediction for design exploration. We note this integration as future work to provide mechanistic context for the high-performance behavior of mix S8-1, A and related eco concretes [105,106,107].

## 5. Conclusions

This study deals with a green, or eco-friendly, concrete with a skeleton formed from coarse aggregates, derived from the recycling of glass waste, which demonstrates once again that through intelligent design, it can achieve high compressive strengths.

The incorporation of SCMs, namely mineral admixtures such as SF and WGP, leads to notable improvements in the hardening properties of concrete, both mechanical and durability, placing ECCs in the HPC category.

SCMs (SF and WGP) exhibit pozzolanic properties that contribute to the refinement of the pore structure, thus improving mechanical properties and resistance to alkali-silica reactions (ASR).

For ECCs containing WGP, after 28 days of curing, the rate of increase in compressive strength is reduced; however, ECCs with the highest WGP content have the highest abrasion resistance.

The evolution of the hardened characteristics of ECCs depends on the nature of the origin of the aggregate, the content of active pozzolanic SCMs and the content of superplasticizer which reduces the water intake from the designed mixture and increases its workability.

The pozzolanic reaction of WGP, but especially SF, with calcium hydroxide (Ca(OH)_2_) leads to the formation of an additional gel, hydrated calcium silicate (C-S-H), known for its essential role in increasing the mechanical characteristics and long-term durability of concrete, as in the present case for the ECC composite, S8-1, A.

The artificial neural network accurately predicted compressive strength based on mixture composition and curing age, demonstrating the effectiveness of shallow feedforward architectures for modeling nonlinear behavior in limited experimental datasets.

The integration of artificial intelligence into the concrete mix design process highlights the potential of machine learning as a decision-support tool, enabling optimization of mixture proportions and performance prediction with reduced reliance on extensive laboratory testing.

## 6. Patents

CONCRETE CONTAINING GLASS WASTE AGGREGATES, RO127399A2 (B1) • 30 May 2012 • UNIV TEHNICĂ DIN CLUJ-NAPOCA [RO], Earliest priority: 15 October 2010 • Earliest publication: 30 May 2012, Inventors, MĂGUREANU CORNELIA [RO]; CORBU OFELIA CORNELIA [RO].

## Figures and Tables

**Table 1:** The physical–mechanical characteristics of CEM I 52.5 R.

Characteristics		UM	Determined Values			Standards
Fineness	The Blaine Method	cm^2^/g	4560 ± 120			SR EN 196-6 [66]
	Sieving method, refusal on 90 mm sieve	%	0			
Determination of setting time and stability	Initial setting time	Min.	130			SR EN 196-3 [67]
	Stability	mm	Max. 10			
Determination of mechanical strengths	Compressive strength 2 days	MPa	33.7	33.9	32.3	SR EN 196-1 [68]
			33.5	32.3	34.1	
			33.3			
	Compressive strength 28 days		63.0	63.0	62.4	
			62.7	62.5	62.0	
			62.6			

**Table 2:** Chemical composition main oxides of OPC, WGP, SP and river sand 0/4 mm.

Composition Main Oxides (%)	Content (wt %)			
	CEM I 52.5 R	SF	WGP	River Sand
SiO_2_	14.30	98.350	74.25	73.60
CaO	69.46	0.334	8.10	0.080
Fe_2_O_3_	3.79	0.168	0.06	0.765
Al_2_O_3_	5.90	0.880	0.25	4.720
SO_3_	3.70	0.035	-	-
Na_2_O	0.07	-	16.27	0.266
MgO	1.30	0.096	0.01	-
B_2_O	-	-	0.39	-
As_2_O_3_			0.20	-
CoO	-	-	0.45	-
LOI	1.48	0.137	0.02	1.200

**Table 3:** Physical properties of aggregates.

Materials	Specific Gravity	Bulk Density	Water Absorption
	g/cm^3^	kg/m^3^	%
River sand 0/4 mm	2.70	1743	3.50
CRA 4/8 mm	2.70	1635	2.40
CRA 8/16 mm	2.70	1635	1.80
CAC 4/8 mm	2.65	1630	1.40
CAC 8/16 mm	2.67	1630	1.20
WGA 0/4 mm	2.47	1640	2.74
WGA 4/8 mm	2.47	1533	0.35
WGA 8/16 mm	2.47	1533	0.30

**Table 4:** ECCs mixture proportions.

Mix	Mixture Proportions												
	CEM I 52.5 R	SF 10%	WGP <0.125 20–30%	^2^ NRA 0/4 45%	WGA 0/4 45%	WGA 4/8 30%	WGA 8/16 25%	Water	^1^ Admixture ACE 30	Density	W/C	W/B	Stages
	kg/m^3^	kg/m^3^	kg/m^3^	kg/m^3^	kg/m^3^	kg/m^3^	kg/m^3^	l/m^3^	l/m^3^	kg/m^3^			
^4^ S7	465	46.5	-	749	-	500	416	153.5	9.30	2339	0.35	0.250	I
S8-1	372	46.5	93	767	-	511	426	122.8	7.44	2346	0.35	0.250	III
S8-2	465	46.5	-	-	716	477	398	153.5	9.30	2265	0.35	0.250	
S8-3	372	46.5	93	-	744	496	413	122.8	7.44	2295	0.35	0.250	
S8-1, A	372	46.5	93	761	-	507	423	125.9	9.30	2338	0.36	0.260	IV
S8-1, B	372	-	139.5	761	-	507	423	125.9	9.30	2337	0.36	0.260	
				NRA 0/4		CRA 4/8	CRA 8/16						II
^4^ Mr	372	46.5	-	820	-	540	457	122.8	7.44	2376	0.35	0.311	
				NRA 0/4		^3^ CAC 4/8	^3^ CAC 8/16						
^4^ M	372	46.5	-	810		540	450	122.8	7.44	2349	0.35	0.311	

^1^ The additive plus the amount of water is included in the calculation of the W/C ratio; ^2^ 0/4 mm Natural River Aggregate for all the mixes (NRA); ^3^ 4/8 mm, 8/16 mm Crushed River Aggregates (CRA); ^4^ S7 is the benchmark mixture of the seven mixtures designed in stage I and Mr and M are control mixtures, made with crushed river aggregates (CRA) and quarry crushed aggregates (CAC)//Crushed aggregates/Chippings (CAC), respectively. All SCMs calculations are related to the initial cement quantity of 465 kg/m^3^.

**Table 5:** ANN Hyperparameters.

Component	Setting
Architecture	Feedforward ANN, 3 hidden layers
Hidden units	32, 16, 8
Activations	ReLU (hidden), linear (output)
Optimizer	Adam
Learning rate	0.001
Batch size	8
Epochs	Up to 3000 with early stopping (patience 100)
Loss	MSE
Regularization	Early stopping used
Input scaling	Standardization

**Table 6:** Hardened concrete properties.

Properties		ECCs							
		S7	S8-1	S8-2	S8-3	S8-1, A	S8-1, B	M	Mr
^1^ f_cm_	7 days	61.1	66.3	59.2	69.1	71.9	48.4	80.4	74.2
	28 days	69.6	70.3	68.0	79.6	83.0	50.3	87.9	79.7
	56 days	76.5	82.9	73.8	79.6	83.7	50.7	92.8	80.3
	90 days	79.9	83.2	74.4	83.7	85.6	52.3	101.7	80.7
	120 days	86.8	85.5	74.7	89.9	88.4	54.2	116.5	89.6
^2^ f_ct,fl_	270 days	4.0	3.1	2.7	3.2	5.8	-	-	-
^3^ f_ct,sp_	28 days	3.7	6.1	4.5	4.5	5.7	-	3.7	1.7
MOE (GPa)	28 days	47	50	50	52	54	-	48	-
Abrasion (mm^3^/5000 mm^2^)		8482	7684	12,216	14,204	7517	5632	6913	7068
Strength loss η (%) at G100		-	-	-	-	5.54	9.87		5.52
Permeability at 12 atm. (mm)		-	-	-	-	10	-	-	-
Carbonation (mm)	360 days	0	0	0	0	0	0	0	0
Shrinkage (mm/m) 120 days		0.20				0.30			

^1^ f_cm_—the average compressive strength of concrete at curing age; ^2^ f_ct,fl_—flexural strength; ^3^ f_ct,sp_ splitting tensile strength in accordance with SR EN 12390-6; G100—samples were subjected to 100 freeze–thaw cycles.

**Table 7:** Model comparison on the held-out test set.

Model	R^2^	MAE (MPa)	RMSE (MPa)
ANN	0.968	1.96	2.52
Random Forest (RF)	0.932	2.40	3.10
SVR (RBF)	0.905	2.85	3.50
Multiple Linear Regression (MLR)	0.800	4.10	5.10

**Table 8:** Grouped cross-validation by mixture.

Model	R^2^ (Mean ± SD)	MAE (MPa) (Mean ±SD)	RMSE (MPa) (Mean ± SD)
ANN	0.920 ± 0.030	2.40 ± 0.40	3.10 ± 0.50
Random Forest (RF)	0.890 ± 0.060	2.75 ± 0.50	3.45 ± 0.60
SVR (RBF)	0.860 ± 0.070	3.05 ± 0.55	3.80 ± 0.65
Multiple Linear Regression (MLR)	0.740 ± 0.100	4.30 ± 0.75	5.15 ± 0.85

**Table 9:** ANN noise sensitivity under grouped CV by mixture.

Noise on Standardized Inputs (σ)	R^2^ (Mean ± SD)	ΔR^2^	MAE (MPa) (Mean ± SD)	ΔMAE (MPa)	RMSE (MPa) (Mean ± SD)	ΔRMSE (MPa)
0.00 (baseline)	0.920 ± 0.030	-	2.40 ± 0.40	-	3.10 ± 0.50	-
0.01	0.916 ± 0.032	−0.004	2.46 ± 0.41	+0.06	3.17 ± 0.51	+0.07
0.02	0.910 ± 0.034	−0.010	2.52 ± 0.43	+0.12	3.25 ± 0.53	+0.15
0.03	0.902 ± 0.036	−0.018	2.60 ± 0.45	+0.20	3.35 ± 0.55	+0.25

**Table 10:** Relations used to determine characteristic strength.

Reference	Formula	Maximum Concrete Class
fib (Buletin 42) [97]	^1^ f_cm_ = f_ck_ + Δf, Δf = 8 MPa	C120/140
NE 012-1/(SR EN 206-1) [69,70]	f_cm_ = ^2^ f_ck_ + (6–12) MPa	C105/115

^1^ f_cm_—the average compressive strength of concrete at a certain age. ^2^ f_ck_, cub—the characteristic compressive strength of concrete determined by testing cubic specimens measuring 150 mm × 150 mm × 150 mm.

**Table 11:** ECCs—Strength class achieved.

Compressive Strength	ECCs							
	S7	S8-1	S8-2	S8-3	S8-1, A	S8-1, B	M	Mr
f_cm_ 28 days	69.6	70.3	68.0	79.6	83.0	50.3	87.9	79.7
f_k_	61.6	62.0	60.0	71.6	75.0	42.3	79.9	71.7
Strength class achieved/concrete-class cubic specimens	C50/60	C50/60	C50/60	C50/60	C60/75	C30/37–C35/45	C60/75	C55/67

**Table 12:** Evolution of the compressive strength of ECCs, Control M, and S8-1, A.

ECCs	Average Compressive Strength of Concrete at Curing Age				
	f_cm_ 7 Days	f_cm_ 28 Days	f_cm_ 56 Day	f_cm_ 90 Days	f_cm_ 120 Days
S8-1, A	71.9	83.0	83.7	85.6	88.4
M	80.4	87.9	92.8	101.7	116.5
S8-1, A/M	0.89	0.94	0.90	0.84	0.76

## Data Availability

The original contributions presented in this study are included in the article. Further inquiries can be directed to the corresponding authors.

## Abbreviations

The following abbreviations are used in this manuscript:

ECC	Engineered Cementitious Composite
WGP	Waste Glass Powder
SF	Silica Fume
SCMs	Supplementary Cementitious Materials
HPC	High-Performance Concrete
CEM	Cement
WGA	Waste Glass Aggregates
ANNs	Artificial Neural Networks
AI	Artificial Intelligence
ML	Machine Learning
